# Supplementary material for: Investigating the Variation in Leaf Traits Within the Allium prattii C.H. Wright Population and Its Environmental Adaptations
Source: Plants (Basel). 2025 Feb 10;14(4):541. doi: 10.3390/plants14040541 (PMC11859293; doi:10.3390/plants14040541)
Supplement: Supplementary file 1 [file plants-14-00541-s001.zip › plants-3394374-supplementary.pdf]

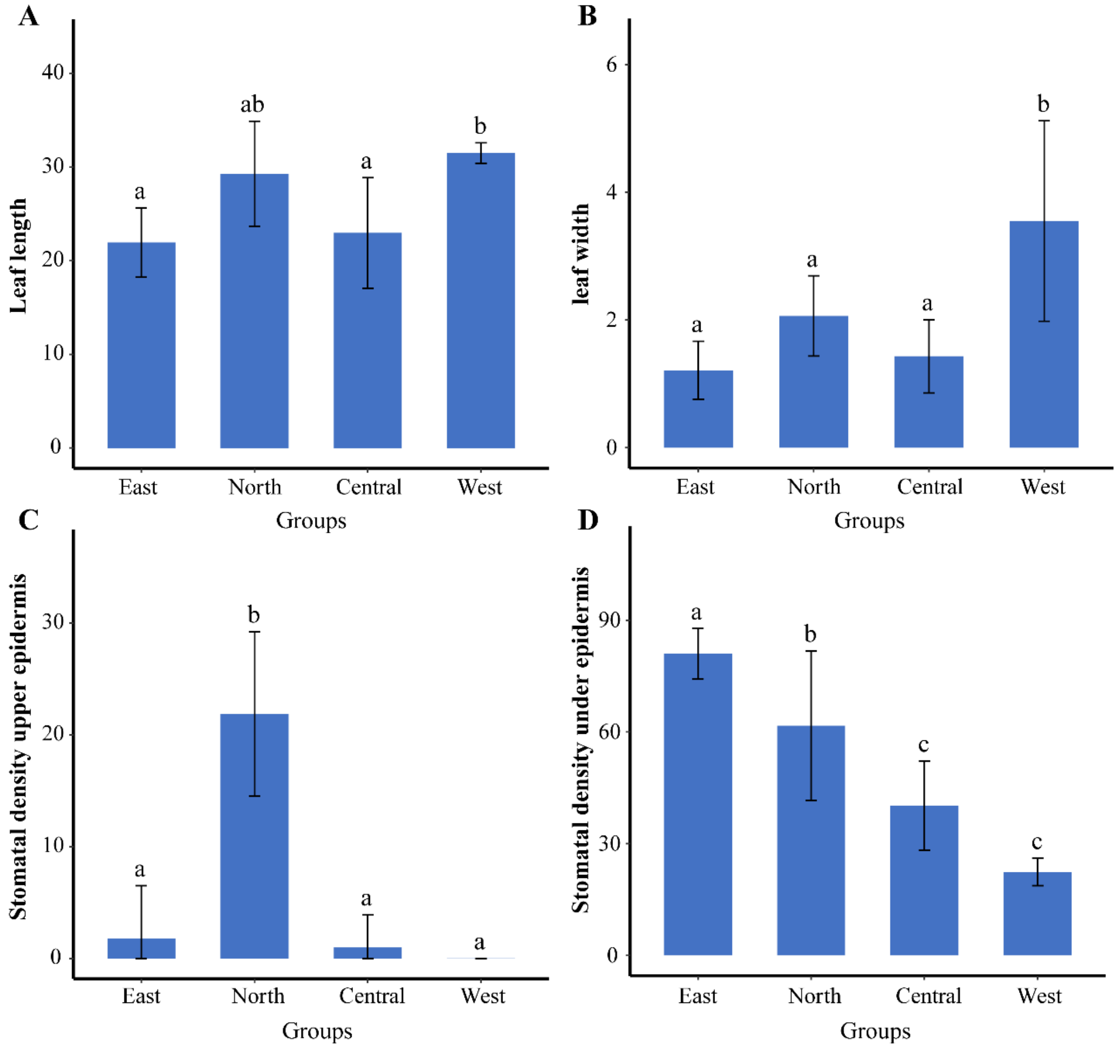


**Figure S1.** ANOVA test for morphological differences among different groups of *A. prattii*. (A) Leaf length; (B) leaf width; (C) stomatal density on the upper leaf epidermis; and (D) stomatal density on the lower leaf epidermis. The significance of morphological differences among groups is indicated by letters a, b, and c, with different letters representing significant differences.

**Table S1. The sample information for 45 populations of *A. prattii*.**

| Number | Location | Population ID | Longitude (E°) | Latitude (N°) | Altitude (m) |
|---|---|---|---|---|---|
| 1 | Huyi, Shanxi | A01 | 108.63 | 33.84 | 2452 |
| 2 | Taibai, Shanxi | A02 | 107.85 | 34.01 | 3429 |
| 3 | Shennonggu, Hubei | A03 | 110.31 | 31.51 | 2913 |
| 4 | Shennongding, Hubei | A04 | 110.3 | 31.45 | 1543 |
| 5 | Wangcang, Sichuan | A05 | 106.62 | 32.74 | 1655 |
| 6 | Maerkang, Sichuan | A06 | 102.75 | 31.92 | 4067 |
| 7 | Dege, Sichuan | A07 | 99.37 | 31.91 | 4332 |
| 8 | Shiqu, Sichuan | A08 | 99.19 | 32.53 | 4395 |
| 9 | Shiqu01, Sichuan | A09 | 98.34 | 33.17 | 4023 |
| 10 | Yushu, Qinghai | A10 | 97.37 | 32.81 | 3997 |
| 11 | Xiaosumang, Qinghai | A11 | 97.34 | 32.51 | 4249 |
| 12 | Chenduo, Qinghai | A12 | 97.22 | 32.71 | 4212 |
| 13 | Nangqian, Qinghai | A13 | 96.34 | 32.33 | 4121 |
| 14 | Leiwuqi, Xizang | A14 | 96.56 | 31.19 | 4365 |
| 15 | Basu, Xizang | A15 | 97.35 | 30.22 | 4556 |
| 16 | Basu01, Xizang | A16 | 96.74 | 29.76 | 4463 |
| 17 | Linzhi, Xizang | A17 | 94.71 | 29.63 | 4502 |
| 18 | Gongbujiangda, Xizang | A18 | 93.45 | 29.96 | 3364 |
| 19 | Mozhugongka, Xizang | A19 | 92.32 | 29.85 | 4765 |
| 20 | Zuogong, Xizang | A20 | 98.38 | 29.72 | 4659 |
| 21 | Xinduqiao Xizang | A21 | 101.21 | 30.24 | 3095 |
| 22 | Yajiang, Sichuan | A22 | 100.92 | 30.61 | 3686 |
| 23 | Litang, Sichuan | A23 | 100.14 | 30.17 | 4023 |
| 24 | Xiangcheng, Sichuan | A24 | 100.32 | 30.62 | 4119 |
| 25 | Daocheng, Sichuan | A25 | 104.27 | 29.15 | 4839 |
| 26 | Xinlong, Sichuan | A26 | 100.32 | 30.35 | 3768 |
| 27 | Yajiang01, Sichuan | A27 | 100.86 | 30.19 | 3942 |
| 28 | Jiulong, Sichuan | A28 | 101.55 | 29.73 | 3374 |
| 29 | Jilong, Xizang | A29 | 85.22 | 28.65 | 4001 |
| 30 | Nielamu, Xizang | A30 | 86.81 | 28.22 | 3843 |
| 31 | Pingdingshan, Henan | A31 | 112.34 | 33.73 | 2116 |
| 32 | Zhaojue, Sichuan | A32 | 102.51 | 27.93 | 3190 |
| 33 | Xianggelila, Yunan | A33 | 99.95 | 28.22 | 4392 |
| 34 | Deqing, Yunnan | A34 | 99.37 | 28.45 | 4403 |
| 35 | Gongshan, Yunnan | A35 | 98.86 | 28.17 | 3884 |
| 36 | Mangkang, Xizang | A36 | 98.73 | 29.32 | 4255 |
| 37 | Mangkang01, Xizang | A37 | 98.66 | 29.93 | 4184 |
| 38 | Batang, Sichuan | A38 | 99.41 | 29.94 | 4750 |
| 39 | Xiangcheng01, Sichuan | A39 | 99.65 | 29.12 | 3805 |
| 40 | Xiangcheng02, Sichuan | A40 | 99.93 | 28.83 | 3250 |
| 41 | Daocheng01, Sichuan | A41 | 100.32 | 28.74 | 3910 |

| | | | | | |
|---|---|---|---|---|---|
| 42 | Bowa mountain, Sichuan | A42 | 100.32 | 28.97 | 4648 |
| 43 | Shade, Sichuan | A43 | 101.72 | 29.54 | 3983 |
| 44 | Kangding, Sichaun | A44 | 101.81 | 30.17 | 4322 |
| 45 | Kangding01, Sichuan | A45 | 102.61 | 29.93 | 3971 |

**Table S2. The extracted average bio-climatic factors values for 45 populations of *A. prattii*.**

| Population ID | Elevation (Alt.)(m) | Isothermality (Bio3) (×100) | Temperature seasonality (Bio4) (standard deviation ×100) | Annual temperature range (Bio7)(℃) | Mean temperature of warmest quarter (Bio10) (℃) | Annual precipitation (Bio12) (mm) | Precipitation of the driest month (Bio14) (mm) |
|---|---|---|---|---|---|---|---|
| A01 | 2452 | 25.83 | 797.63 | 29.20 | 14.43 | 820.00 | 7.00 |
| A02 | 3429 | 25.47 | 769.28 | 28.30 | 10.05 | 858.00 | 8.00 |
| A03 | 2913 | 26.57 | 753.45 | 28.20 | 13.17 | 1366.00 | 23.00 |
| A04 | 1543 | 27.00 | 754.18 | 28.30 | 13.40 | 1373.00 | 24.00 |
| A05 | 1655 | 26.58 | 756.72 | 28.00 | 18.48 | 919.00 | 8.00 |
| A06 | 4067 | 39.34 | 657.14 | 31.50 | 8.32 | 752.00 | 4.00 |
| A07 | 4332 | 37.02 | 745.45 | 32.80 | 7.12 | 633.00 | 6.00 |
| A08 | 4395 | 40.81 | 728.30 | 34.00 | 8.02 | 611.00 | 5.00 |
| A09 | 4023 | 41.62 | 763.06 | 35.80 | 8.72 | 534.00 | 4.00 |
| A10 | 3997 | 40.73 | 769.38 | 35.60 | 9.45 | 534.00 | 3.00 |
| A11 | 4249 | 39.65 | 755.77 | 34.30 | 7.08 | 591.00 | 5.00 |
| A12 | 4212 | 38.96 | 760.69 | 34.20 | 7.60 | 577.00 | 4.00 |
| A13 | 4121 | 41.02 | 721.43 | 33.50 | 9.40 | 572.00 | 3.00 |
| A14 | 4365 | 40.30 | 698.34 | 31.80 | 8.67 | 597.00 | 4.00 |
| A15 | 4556 | 44.33 | 648.44 | 31.30 | 8.55 | 587.00 | 4.00 |
| A16 | 4463 | 40.92 | 669.53 | 30.30 | 7.40 | 656.00 | 6.00 |
| A17 | 4502 | 39.08 | 641.59 | 28.70 | 7.78 | 625.00 | 5.00 |
| A18 | 3364 | 41.33 | 631.64 | 29.40 | 13.45 | 504.00 | 2.00 |
| A19 | 4765 | 40.51 | 692.56 | 31.80 | 6.20 | 423.00 | 3.00 |
| A20 | 4659 | 43.94 | 628.11 | 30.00 | 6.30 | 611.00 | 5.00 |
| A21 | 3095 | 42.87 | 627.88 | 29.80 | 14.08 | 686.00 | 1.00 |
| A22 | 3686 | 43.39 | 602.21 | 29.50 | 10.58 | 689.00 | 1.00 |
| A23 | 4023 | 45.90 | 629.53 | 31.90 | 9.50 | 601.00 | 1.00 |
| A24 | 4119 | 47.68 | 623.39 | 32.30 | 8.63 | 621.00 | 1.00 |
| A25 | 4839 | 25.39 | 696.39 | 25.90 | 25.95 | 1325.00 | 17.00 |
| A26 | 3768 | 43.37 | 626.33 | 30.30 | 10.25 | 627.00 | 1.00 |
| A27 | 3942 | 44.11 | 618.14 | 30.40 | 9.87 | 675.00 | 1.00 |
| A28 | 3374 | 44.65 | 607.38 | 29.90 | 12.43 | 740.00 | 1.00 |
| A29 | 4001 | 47.22 | 525.23 | 26.40 | 9.08 | 455.00 | 11.00 |
| A30 | 3843 | 47.89 | 523.85 | 26.40 | 10.92 | 675.00 | 14.00 |
| A31 | 2116 | 28.51 | 820.62 | 30.90 | 17.43 | 937.00 | 11.00 |
| A32 | 3190 | 45.19 | 513.05 | 25.10 | 13.53 | 925.00 | 5.00 |
| A33 | 4392 | 42.07 | 588.35 | 26.90 | 7.00 | 711.00 | 7.00 |
| A34 | 4403 | 40.25 | 617.59 | 27.10 | 7.45 | 643.00 | 7.00 |

| | | | | | | | |
|---|---|---|---|---|---|---|---|
| A35 | 3884 | 37.11 | 589.53 | 24.70 | 8.67 | 717.00 | 12.00 |
| A36 | 4255 | 39.47 | 637.97 | 28.10 | 8.83 | 571.00 | 5.00 |
| A37 | 4184 | 47.92 | 603.45 | 31.20 | 9.93 | 578.00 | 3.00 |
| A38 | 4750 | 43.58 | 630.63 | 30.10 | 7.85 | 596.00 | 3.00 |
| A39 | 3805 | 40.57 | 631.25 | 28.80 | 10.57 | 591.00 | 3.00 |
| A40 | 3250 | 42.98 | 614.70 | 29.30 | 13.63 | 624.00 | 3.00 |
| A41 | 3910 | 43.35 | 614.79 | 29.70 | 10.23 | 662.00 | 3.00 |
| A42 | 4648 | 44.73 | 592.44 | 29.40 | 6.90 | 686.00 | 3.00 |
| A43 | 3983 | 44.90 | 600.24 | 29.90 | 9.73 | 778.00 | 2.00 |
| A44 | 4322 | 42.92 | 628.99 | 30.60 | 7.83 | 750.00 | 3.00 |
| A45 | 3971 | 41.36 | 615.65 | 29.50 | 9.12 | 772.00 | 3.00 |

**Table S3. The AUC values of the prediction models.**

| Period | Training data | Test data |
|---|---|---|
| LIG | 0.975±0.006 | 0.973±0.007 |
| LGM | 0.968±0.009 | 0.974±0.007 |
| MH | 0.967±0.006 | 0.959±0.016 |
| Current | 0.971±0.007 | 0.968±0.009 |
| 2021-2040，ssp245 | 0.973±0.006 | 0.968±0.013 |
| 2041-2060，ssp245 | 0.965±0.007 | 0.956±0.015 |

**Table S4. the contribution and importance of bioclimatic factors in prediction models.**

| Variable | Percent contribution | Permutation importance |
|---|---|---|
| Alt | 5.8 | 2 |
| Bio4 | 44.9 | 15.1 |
| Bio10 | 30.8 | 30 |
| Bio12 | 8 | 10.8 |
| Bio7 | 6.8 | 38.6 |
| Bio3 | 2.5 | 2.6 |
| Bio14 | 1.3 | 0.9 |

**Table S5. The 19 bioclimatic factors used in this study.**

| Bioclimatic factor | Description |
|---|---|
| Alt* | Altitude |
| Bio1 | Annual Mean Temperature |
| Bio2 | Mean Diurnal Range (Mean of monthly (max temp - min temp)) |
| Bio3* | Isothermality (BIO2/BIO7) (×100) |
| Bio4* | Temperature Seasonality (standard deviation ×100) |
| Bio5 | Max Temperature of Warmest Month (℃) |
| Bio6 | Min Temperature of Coldest Month (℃) |
| Bio7* | Temperature Annual Range (BIO5-BIO6) (℃) |
| Bio8 | Mean Temperature of Wettest Quarter (℃) |
| Bio9 | Mean Temperature of Driest Quarter (℃) |
| Bio10* | Mean Temperature of Warmest Quarter (℃) |
| Bio11 | Mean Temperature of Coldest Quarter (℃) |
| Bio12* | Annual Precipitation (mm) |
| Bio13 | Precipitation of Wettest Month (mm) |
| Bio14* | Precipitation of Driest Month (mm) |
| Bio15 | Precipitation Seasonality (Coefficient of Variation) (mm) |
| Bio16 | Precipitation of Wettest Quarter (mm) |
| Bio17 | Precipitation of Driest Quarter (mm) |
| Bio18 | Precipitation of Warmest Quarter (mm) |
| Bio19 | Precipitation of Coldest Quarter（(mm) |

*: indicates the climatic factors ultimately selected in this study.
